# Effectiveness of the FED Method in the Treatment of Idiopathic Scoliosis of Girls Aged 11–15 Years

**DOI:** 10.3390/ijerph19010065

**Published:** 2021-12-22

**Authors:** Sandra Trzcińska, Kamil Koszela, Michał Kuszewski

**Affiliations:** 1 Department of Physiotherapy, College of Rehabilitation in Warsaw, 01-234 Warsaw, Poland; sandra-trzcinska@wp.pl; 2 Neuroorthopedics and Neurology Clinic and Polyclinic, National Institute of Geriatrics, Rheumatology and Rehabilitation, 02-637 Warsaw, Poland; 3 Institute of Physiotherapy and Health Sciences, Academy of Physical Education, 40-065 Katowice, Poland; m.kuszewski@awf.katowice.pl

**Keywords:** idiopathic scoliosis, scoliosis, rehabilitation, spine, conservative treatment, musculoskeletal disorders, FED, FITS

## Abstract

(1) Background: The unknown etiology of idiopathic scoliosis and its three-dimensional nature make the cause-and-effect therapeutic management difficult. A tendency to progression of scoliosis and the failure of many methods of conservative treatment have prompted the search for new methods that would stop and correct deformations. One of them is the FED method, used in the conservative treatment of idiopathic scolioses, in which all scoliotic curves are corrected. The aim of this study was a comparative analysis of the effectiveness of idiopathic scoliosis treatment with the FED and FITS methods. (2) Methods: The study included 60 randomly selected girls, aged 11 to 15 years, treated with the FED and FITS methods. They were diagnosed with idiopathic scoliosis grade II according to Cobb and double-curve scoliosis type I and II according to King–Moe classification. The results of the therapy were assessed with the use of the Bunnell scoliometer. The examinations were performed before the start of the therapy—on the first day of the child’s stay—and 3 weeks after the therapy. The angle of trunk rotation and the sum of two rotations were assessed using a scoliometer. (3) Results: The performed statistical analysis demonstrated significant changes in the examined parameters in both therapeutic groups. (4) Conclusions: 1. The obtained results indicate that the FED therapy may prove to be an effective method of treating idiopathic scoliosis; however, it requires further research in a larger group of patients; 2. both methods significantly improved trunk rotation in primary and secondary scoliosis, but after using summing parameters (SDR parameter), the FED method appeared to be statistically more effective.

## 1. Introduction

Scoliosis is a curvature of the spine with an angle of at least 10° as measured by the Cobb method on a radiograph in the frontal plane. It is a three-dimensional deformation. Idiopathic scoliosis is one of the most difficult problems in modern orthopedics and physiotherapy. It accounts for 80–90% of all types of scolioses [1]. Its unknown etiology and three-dimensional nature make the cause-and-effect therapeutic management difficult [2]. A tendency to progression of scolioses and the failure of many methods of conservative treatment have resulted in the search for new methods that would stop and correct deformities [3]. One of them is the FED method (fixation, elongation, derotation) used in the conservative treatment of idiopathic scoliosis [4]. The FED treatment method uses a device where corrective forces act on the curvature. The strength of the device is focused on stabilizing, stretching, and derotating the spine, under the control of an innovative computer program. It has been operating in Poland since 2010. A commonly used method of treating scoliosis in Poland is the FITS method, defined as a comprehensive, asymmetric, individual therapy. There is no device in the FITS method. It was created in 2004.

It includes the correction of all scoliotic curves, as opposed to other therapeutic methods that can cause the phenomenon of reducing the primary curve at the expense of increasing the secondary ones. This mechanism is especially visible when examining the rotation of the trunk with the Bunnell scoliometer, that is why the sum of individual parameters defining the global value of the rotation of the whole spine was used to assess the effects of idiopathic scolioses therapy. The correlation of the trunk rotation angle with the Cobb angle may indicate that the reduction of the costal hump is equivalent to obtaining the correction of the deformation [5]. Therefore, taking into account the harmfulness of X-ray radiation and the limited possibility of a frequent radiological control of the spinal curvature, a scoliometer has become a common tool in the assessment of treatment results in patients with idiopathic scoliosis [6,7].

Informing scientists about FED methods may increase interest in this type of treatment, which is still little known and may turn out to be as effective as the FITS method recommended by SOSORT.

The aim of this study was a comparative analysis of the effectiveness of idiopathic scolioses treatment with the FED and FITS methods (functional individual therapy of scolioses) of girls aged 11 to 15 years.

Hypothesis: The FED method of treating idiopathic scoliosis in girls aged 11 to 15 years is more effective than the FITS method.

## 2. Materials and Methods

On the basis of the analysis of X-ray scans and the inclusion and exclusion criteria, 60 girls aged 11 to 15 years (mean 13.58 ± 1.33) were randomly selected for the study. They were diagnosed with idiopathic scoliosis II° according to Cobb (the value of the primary curve was in the range 30–60°) and double-curve scoliosis of type I and II according to the King–Moe classification. 

The patients were diagnosed by an orthopedist who diagnosed idiopathic scoliosis on the basis of an X-ray image and the patient’s medical history. 

The examination and treatment were carried out by the same doctor.

The girls participated in a 3-week rehabilitation program. The patients were additionally provided with a Boston type brace.

Inclusion criteria:

- current X-ray scan covering the pelvic girdle, diagnosed double-curve idiopathic scoliosis (type I and II acc. to King–Moe classification), with the Cobb angle between 30 and 60° of primary scoliosis
- girls‘ age, 11–15 years
- unfinished ossification with Risser sign below 5
- no contraindications to the therapy from other systems
- a consent to the examination procedures

Exclusion criteria:

- congenital spine defect preventing the initiation of the therapy, neurological deficits and cerebral palsy and significantly impaired sight and/or hearing
- presence of the third curve in the cervico-thoracic spine, reaching the fourth thoracic vertebra, manifested as a rotation exceeding 7° at the level of the 7th cervical vertebra as measured with a scoliometer, and the Cobb angle in the X-ray scan greater than 10°.

The girls were randomly assigned to two groups: treated with the FED method, and the other treated with the FITS method. The comparison of the two groups in terms of age and X-ray analysis indicators demonstrated no significant changes in the analyzed parameters (Table 1).

Rehabilitation using the FED method was carried out in the ward in the morning hours, while FITS rehabilitation was carried out in the afternoon, depending on the organization of work in the department. Some of the patients could only come in the morning, and some in the afternoon. In this way, the patients were randomly assigned to particular groups.

The type of scoliosis was also not different between the two studied groups (*p* > 0.05). Detailed data are presented in Table 2.

Each girl underwent initial orthopedic examination, which included a radiological examination (X-ray) performed in the anteroposterior position. Cobb angles and Raimondi rotation were measured for primary and secondary scoliosis. The skeletal maturity was evaluated with the Risser sign. The results of the therapy were assessed with the use of the Bunnell scoliometer. The examinations were performed before the start of the therapy—on the first day of the child’s stay—and after 3 weeks—on the last day of the rehabilitation stay. Each of the examined patients could withdraw from the therapy at any time. In order to assess the trunk rotation angle, the Adams test was performed using the Bunnell scoliometer, which is a simple and convenient measuring device allowing the rapid assessment of trunk rotation. It is characterized by high sensitivity and repeatability [8]. From a relaxed standing position with straight legs and feet hip-width apart, the examined girls bent their trunk forward. The scoliometer was placed perpendicularly to the trunk, transversely to the long axis of the spine, above the line of the spinous processes. The scale of the scoliometer showed the value of rotation in degrees consecutively for each vertebra from C7 to L5 with an accuracy of 1°. To eliminate the error, the measurement was performed twice, and the final rotation value resulted from their mean value. When assessing the apexes, the area with the highest rotation value was selected, for both primary and secondary scoliosis for the cervicothoracic, thoracic, and lumbar sections. The angle of trunk rotation and the sum of two rotations were assessed using a scoliometer.

The measurement of the angle of trunk rotation (ATR) was performed at the apex of the most rotated vertebra of the thoracic and lumbar spine, with the determination of the primary scoliosis (ATR P parameter) and the secondary scoliosis (ATR W). To analyze the behavior of the cervicothoracic section, the rotation test was performed on the spinous process of the last cervical vertebra—ATR C. If the value of rotation measured with the Bunnell’s scoliometer was greater than 7°, the curve was considered scoliotic [9,10]. The presence of this curve excluded the patient from the study. 

The measurement of the sum of two rotations (SDR) consisted in summing two values of the angle of trunk rotation, i.e., at the apex of the thoracic curve and at the apex of the lumbar spine. The values of rotation measured with the accuracy of 1° of left-sided and right-sided curves were summed up as positive values. A simplification of the measurement provided an overview of the behavior of both sections—primary and secondary—and represented an evaluation of the global value of trunk rotation in double-curve scoliosis.

The girls were randomly assigned to two groups of therapeutic strategies.

The study group included 30 patients treated with the FED method according to its assumptions [11]. In each patient, two physiotherapeutic procedures were performed: electrostimulation and thermotherapy with the use of thermogels, which prepared them for the therapy in the FED device. Then, the girls performed asymmetrical exercises, which were selected individually on the basis of the King–Moe classification of scoliosis. 

The control group included 30 patients treated with the FITS method as the Polish recommended method of conservative treatment of patients with scoliosis [12]. The therapy program included educating the patient about their deformation, learning ways of self-correction of the deformity with proper foot loading, stabilization of the lower lumbar spine, learning corrective tension, development of new corrective postural patterns in functional positions, correction of the primary curve by adding functional compensation [13,14].

The study was approved by the Bioethics Committee for Scientific Research at the Jerzy Kukuczka Academy of Physical Education in Katowice, No: 10/2013. The study was conducted according to the guidelines of the Declaration of Helsinki. Written consent was obtained for the study.

### Statistical Analysis

The Statistical Package for the Social Sciences (SPSS, Fisioconsum S.L., Chicago, IL, USA) was used to perform statistical Analyses. The Shapiro–Wilk test was used to check whether the distribution of the quantitative parameters in the investigated sample differed from the theoretical normal distribution. Therefore, the following non-parametric statistical methods were used to verify the research hypotheses: the Mann–Whitney rank-sum U-test for comparing the distributions of quantitative variables, the Wilcoxon signed-rank test for dependent groups to compare the distributions of quantitative variables, the Pearson’s chi-square test (χ2 test of independence) for comparing the distributions of qualitative variables. The level of significance was α = 0.05 for the verification of the statistical hypotheses (statistically significant results are marked in bold).

## 3. Results

The performed statistical analysis demonstrated significant changes in the examined parameters for both therapeutic groups. Detailed data are presented in Table 3, Table 4 and Table 5.

A statistically significant change was found between the measurements of the cervicothoracic rotation angle (ATR C), at the apex of the primary (ATR P) and secondary (ATR W) scoliosis, in both groups of girls treated with the FED and FITS methods. Detailed data are presented in Table 6, Table 7 and Table 8.

The analysis revealed statistically significant differences between the groups for the value of rotation at the apex of the primary scoliosis. However, no statistically significant differences were found between the groups in terms of rotation angle change at the level of the cervicothoracic spine and at the apex of the secondary scoliosis after the therapy. Detailed data are presented in Table 9 and Table 10.

A statistically significant improvement was found between the measurements of the sum of two rotations, in both groups of girls treated with the FED and the FITS methods.

Scoliometer examination, change in SDR parameter:

We demonstrated statistically significant differences between the groups for the investigated variable.

## 4. Discussion

The FED method is a relatively new method of conservative treatment of idiopathic scolioses in Poland. Due to the high cost of the device, the number of centers treating scoliosis using the FED method is still scarce. This has a huge impact on the number of related publications [15,16]. However, the largest research material was presented by the creator of the method, Prof. Sastre [17,18]. A small number of studies on the FED method makes the research on the evaluation of its effectiveness unique and largely based on individual experiences. However, this limits the possibility of comparing the obtained results with other studies, though, at the same time, it encourages scientists to further explore the subject. 

During the period of intensive growth, spinal deformity may progress, with a marked predominance of girls in the manifestation of scoliosis [19,20]. Therefore, in order to objectify the assessment of the undertaken treatment methods, girls aged 1–15 years with incomplete bone growth were enrolled in the study. 

The use of bone maturity assessment, Cobb angle, and rotation in radiological analysis is important in predicting the final results of a therapy [21].

The analysis of posture parameters is very important for health status assessment. 

The measurement of the Cobb angle on a radiograph of the spine is the gold standard in the diagnosis of scolioses. However, the side effects and harmfulness of X-ray radiation limit the frequency of this examination. Therefore, the Bunnell scoliometer is increasingly used to assess the effectiveness of treatment and to monitor changes in the scoliotic deformity [22]. There is also a correlation between the size of the costal hump and the Cobb angle [23]. The conducted study demonstrated a significant improvement in all parameters, in both groups of girls, independently of the treatment received (FED or FITS methods, ATR C, ATR P, and ATR W parameters). Although the patients treated with the FED method showed a greater improvement of the value of rotation measured in the primary scoliosis (ATR P) and secondary scoliosis (ATR W) and of cervicothoracic rotation (ATR C), a statistically significant difference between the groups occurred only for the primary scoliosis. 

The presence of compensatory curves requires the monitoring of spinal deformations including all scoliotic curves. In practice, however, in order to assess the effects of a therapy, the analysis of the parameters related to the correction of the primary curve is mainly used [24,25]. Under the influence of FITS therapy, there is a significant improvement in the main curve, but reports demonstrate a simultaneous tendency of an increased angle of trunk rotation within compensatory curves, as a development of compensatory mechanisms in idiopathic scoliosis resulting from the administered therapy [26]. In the FED method, owing to the stabilization of the arms of the FED device at the level of the secondary curve, both curves are subjected to therapy and are thus reduced. Therefore, after 3 weeks of treatment with the FED method, the correction of all curves was achieved, including the improvement of the global rotation of the trunk. In patients with double-curve scoliosis, measurements can be made in two places of maximal rotation, i.e., at the level of the thoracic and of the lumbar sections [27]. The statistical decrease in the ATR C value after therapy in both groups may indicate the possibility of using the sum of two rotations without the need to sum up the three levels. Neither method increased the rotation at the level of the cervicothoracic spine. 

In Poland, the summing parameters are still little known; however, Kotwicki et al. [28] used the Sum of Rotation parameter as the sum of rotations at the level of 12 thoracic and 5 lumbar vertebrae, with a correction for pelvic rotations. 

Other authors noted the need to examine the spine at different levels when diagnosing scoliosis, summing the trunk rotations with a scoliometer in the Adams test to monitor treatment as a measure of the total rotational deformation of the trunk [29,30]. A statistical correlation was also demonstrated between the change in the Cobb angle of the main curve and the Hump Sum parameter, which derives from summing up the three values of the surface angle of trunk rotation measured at the level of the thoracic proximal, thoracic middle, and lumbar spine [31]. 

Due to the fact that the research was limited to girls with double-curve scolioses, the parameter of a sum of two rotations (SDR) was used. Summing two levels of rotation provides an overview of the changes in these values within both the primary and the compensatory segment. Therefore, it is worth striving to reduce the SDR parameter with the therapy. Similar results of the reduction of both curves in the treatment of double-curve scoliosis were reported by Czaprowski et al. [32]. Thus, a reduction in the parameter of the sum of two rotations (SDR) will indicate the correction of all curves, while its lack of change or increase will mean the development of compensatory curves, i.e., a reduction in the primary curve at the expense of the increase in the compensatory curve. Analyzing this parameter in the conducted research, a significant improvement was obtained in both groups, but patients treated with the FED method showed statistically greater improvement compared to the control group. 

This study allowed evaluating the effectiveness of two different methods of conservative treatment. It also encourages the introduction of summing parameters for the diagnosis of idiopathic scoliosis. 

The selected inclusion and exclusion criteria regarding the type of scoliosis and the age of the patients offered great opportunities for an objective comparison of the two strategic groups; however, they largely limited the number of patients qualified for the study and thus reduced the number of the examined subjects. Probably, more information on the effectiveness of the FED method in the treatment of idiopathic scolioses would be provided by long-term diagnostics; however, the program-oriented nature of rehabilitation in hospital conditions lasting 3 weeks and covering the treatment of patients from all over Poland limits further examinations after hospitalization. 

The obtained results indicate that the FED therapy is an effective method of treating idiopathic scolioses; however, it requires further analyses supplemented with long-term follow-up in a larger group of patients.

## 5. Conclusions

1. The obtained results indicate that the FED therapy may prove to be an effective method of treating idiopathic scoliosis; however, it requires further research in a larger group of patients.
2. Both assessed methods significantly improved the rotation of the trunk in primary and secondary scoliosis, but after using summing parameters (SDR parameter), the FED method proved to be statistically more effective.

## Figures and Tables

**Table 1:** Age and X-ray analysis indicators in girls undergoing FED and FITS therapies.

Variable	Group	*n*	*x* ± *SD*	*Min*	*Max*	*p*
Age [years]	FED	30	13.53 ± 1.33	11	15	0.788
	FITS	30	13.63 ± 1.35	11	15	
Cobb angle for primary scoliosis [°]	FED	30	42.47 ± 8.59	30	59	0.420
	FITS	30	40.63 ± 8.52	30	60	
Cobb angle for secondary scoliosis [°]	FED	30	33.70 ± 9.56	19	56	0.198
	FITS	30	30.57 ± 8.81	18	51	
Risser sign [score]	FED	30	2.67 ± 1.27	0	4	0.219
	FITS	30	2.87 ± 1.57	0	4	
Raimondi rotation [°]	FED	30	16.70 ± 7.15	2	33	0.468
	FITS	30	18.10 ± 7.22	4	31	

*n*—number, *x*—mean, *SD*—standard deviation, *Min*—the lowest value, *Max*—the highest value, *p*—level of significance.

**Table 2:** Type of scoliosis in girls treated with the FED and FITS methods.

King-Moe Classification	Group				Total	
	FED		FITS			
	*n*	% of the Group	*n*	% of the Group	*n*	% of Total
Type I	8	26.67	7	23.33	15	25.00
Type II	22	73.33	23	76.67	45	75.00
Total	30	100.00	30	100.00	60	100.00

*n*—number.

**Table 3:** Cervicothoracic rotation angle (ATR C) before and after therapy.

Group	Measurement Moment	ATR C [°]				*p*
		n	*x ± SD*	*Min*	*Max*	
FED	Before therapy	30	3.30 ± 1.62	0	6	0.001
	After therapy	30	1.57 ± 1.43	0	5	
	Δ	30	−1.73 ± 1.44	−4	0	
FITS	Before therapy	30	2.20 ± 1.85	0	5	0.001
	After therapy	30	0.83 ± 0.99	0	3	
	Δ	30	−1.37 ± 1.50	−5	0	

*n*—number, *x*—mean, *SD*—standard deviation, *Min*—the lowest value, *Max*—the highest value, Δ—difference, *p*—level of significance, ATR C—cervical vertebra trunk rotation.

**Table 4:** Primary scoliosis cervicothoracic rotation angle (ATR P) before and after therapy.

Group	Measurement Moment	ATR P [°]				*p*
		*n*	*x ± SD*	*Min*	*Max*	
FED	Before therapy	30	12.87 ± 4.95	3	24	0.001
	After therapy	30	9.87 ± 4.13	2	21	
	Δ	30	−3.00 ± 1.95	−8	0	
FITS	Before therapy	30	11.23 ± 5.31	1	23	0.001
	After therapy	30	9.27 ± 4.96	1	22	
	Δ	30	−1.97 ± 1.59	−5	0	

*n*—number, *x*—mean, *SD*—standard deviation, *Min*—the lowest value, *Max*—the highest value, Δ—difference, *p*—level of significance, ATR P—primary trunk rotation.

**Table 5:** Secondary scoliosis cervicothoracic rotation angle (ATR W) before and after therapy.

Group	Measurement Moment	ATR W [°]				*p*
		*n*	*x ± SD*	*Min*	*Max*	
FED	Before therapy	30	7.20 ± 3.55	0	15	0.001
	After therapy	30	4.87 ± 3.14	0	13	
	Δ	30	2.33 ± 1.65	−7	0	
FITS	Before therapy	30	7.70 ± 4.10	2	16	0.001
	After therapy	30	6.00 ± 3.97	0	16	
	Δ	30	−1.70 ± 2.09	−6	1	

*n*—number, *x*—mean, *SD*—standard deviation, *Min*—the lowest value, *Max*—the highest value, Δ—difference, *p*—level of significance, ATR W—secondary trunk rotation.

**Table 6:** Level of rotation angle change at the apex of the cervicothoracic curve (ATR C).

Variable	Group	*n*	*x ± SD*	*Min*	*Max*	*p*
ATR C [°] change	FED	30	−1.73 ± 1.44	−4	0	0.247
	FITS	30	−1.37 ± 1.50	−5	0	

*n*—number, *x*—mean, *SD*—standard deviation, *Min*—the lowest value, *Max*—the highest value, *p*—level of significance, ATR C—cervical vertebra trunk rotation.

**Table 7:** Level of rotation angle change at the apex of the primary cervicothoracic scoliosis (ATR P change).

Variable	Group	*n*	*x ± SD*	*Min*	*Max*	*p*
ATR P [°] change	FED	30	−3.00 ± 1.95	−8	0	0.049
	FITS	30	−1.97 ± 1.59	−5	0	

*n*—number, *x*—mean, *SD*—standard deviation, *Min*—the lowest value, *Max*—the highest value, *p*—level of significance, ATR P—primary trunk rotation.

**Table 8:** Level of rotation angle change at the apex of the secondary cervicothoracic scoliosis (ATR W change).

Variable	Group	*n*	*x ± SD*	*Min*	*Max*	*p*
ATR W [°] change	FED	30	−2.33 ± 1.65	−7	0	0.122
	FITS	30	−1.70 ± 2.09	−6	1	

*n*—number, *x*—mean, *SD*—standard deviation, *Min*—the lowest value, *Max*—the highest value, *p*—level of significance, ATR W—secondary trunk rotation.

**Table 9:** The sum of two rotations (SDR) before and after therapy in girls treated with the FED and FITS methods.

Group	Measurement Moment	SDR [°]				*p*
		*n*	*x ± SD*	*Min*	*Max*	
FED	Before therapy	30	20.07 ± 5.32	11	31	0.001
	After therapy	30	14.73 ± 4.35	8	26	
	Δ	30	−5.33 ± 2.67	−11	0	
FITS	Before therapy	30	18.93 ± 6.64	6	35	0.001
	After therapy	30	15.27 ± 6.26	2	29	
	Δ	30	−3.67 ± 2.22	−8	0	

*n*—number, *x*—mean, *SD*—standard deviation, *Min*—the lowest value, *Max*—the highest value, Δ—difference, *p*—level of significance, SDR—sum of two rotations.

**Table 10:** Level of change in the sum of two rotations (SDR change) after the therapy in girls treated with the FED and FITS methods.

Variable	Group	*n*	*x ± SD*	*Min*	*Max*	*p*
SDR [°] change	FED	30	−5.33 ± 2.67	−11	0	0.012
	FITS	30	−3.67 ± 2.22	−8	0	

*n*—number, *x*—mean, *SD*—standard deviation, *Min*—the lowest value, *Max*—the highest value, *p*—level of significance, SDR—sum of two rotations.

## Data Availability

The datasets analyzed during the current study are available from the corresponding author upon reasonable request.
